# An Expression for Velocity Lag in Sediment-Laden Open-Channel Flows Based on Tsallis Entropy Together with the Principle of Maximum Entropy

**DOI:** 10.3390/e21050522

**Published:** 2019-05-23

**Authors:** Zhongfan Zhu, Jingshan Yu, Jie Dou, Dingzhi Peng

**Affiliations:** 1 Beijing Key Laboratory of Urban Hydrological Cycle and Sponge City Technology, College of Water Sciences, Beijing Normal University, Beijing 100875, China; 2 Department of Civil and Environmental Engineering, Nagaoka University of Technology 1603-1, Kami-Tomioka, Nagaoka 940-2188, Japan

**Keywords:** Tsallis entropy, velocity lag, sediment-laden flow, open channels

## Abstract

In the context of river dynamics, some experimental results have shown that particle velocity is different from fluid velocity along the stream-wise direction for uniform sediment-laden open-channel flows; this velocity difference has been termed velocity lag in the literature. In this study, an analytical expression for estimating the velocity lag in open-channel flows was derived based on the Tsallis entropy theory together with the principle of maximum entropy. The derived expression represents the velocity lag as a function of a non-dimensional entropy parameter depending on the average and maximum values of velocity lag from experimental measurements. The derived expression was tested against twenty-two experimental datasets collected from the literature with three deterministic models and the developed Shannon entropy-based model. The Tsallis entropy-based model agreed better with the experimental datasets than the deterministic models for eighteen out of the twenty-two total real cases, and the prediction accuracy for the eighteen experimental datasets was comparable to that of the developed Shannon entropy-based model (the Tsallis entropy-based expression agreed slightly better than the Shannon entropy-based model for twelve out of eighteen test cases, whereas for the other six test cases, the Shannon entropy-based model had a slightly higher prediction accuracy). Finally, the effects of the friction velocity of the flow, the particle diameter, and the particles’ specific gravity on the velocity lag were analyzed based on the Tsallis entropy-based model. This study shows the potential of the Tsallis entropy theory together with the principle of maximum entropy to predict the stream-wise velocity lag between a particle and the surrounding fluid in sediment-laden open-channel flows.

## 1. Introduction

**Figure 1:**
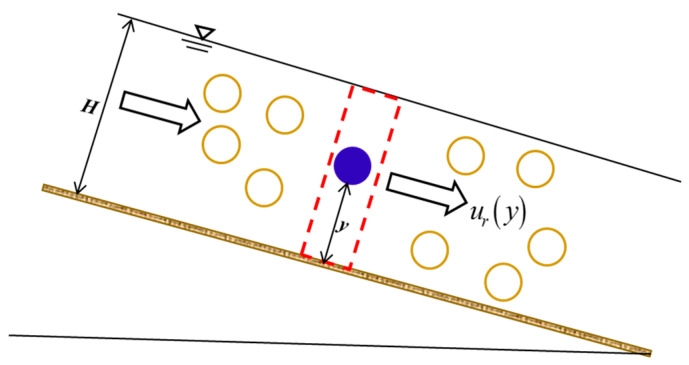
Schematic diagram of the velocity lag between the sediment particle and the surrounding fluid in sediment-laden flow. In this figure, $u_{r}\left(y\right)$ is velocity lag at a vertical distance y from the channel bottom, and H is the water depth of sediment-laden flow, respectively. Here the velocity of the sediment particle is the bulk velocity of the sediment particles (i.e., it is defined statistically as the average velocity of many sediment particles), which is different from the velocity of individual sediment particles.

Many studies have investigated the impact of suspended sediment on fluid velocity distribution from experimental or theoretical viewpoints, but few studies have analyzed the velocity of sediment particles in open-channel turbulent flows [1]. The velocity of sediment particles is an important parameter in determining the suspended sediment transport rate: for example, according to Einstein’s method, the actual suspended load of sediment-laden flows should be calculated by integrating the product of the velocity of sediment particle and the particle concentration [2,3]. Aziz [3] reported that the suspended load calculated by integrating the product of the fluid velocity and the particle concentration was 6–37% greater than the actual load. In sediment-laden flows, it is always assumed that the sediment particle follows the fluid completely along the stream-wise direction, implying that the stream-wise velocity difference between them is equally zero [2]. However, some researchers have reported that the particle velocity is less than that of the carrier fluid; this velocity difference has been termed “velocity lag” in the literature, as illustrated by Figure 1, but it is difficult to quantify, due to the limitations of the measurement instruments (e.g., [3,4]).

The velocity lag problem mainly referred to coarse or fine particles suspended in open-channel flows (that is, suspended load). With the rapid development of advanced equipment, such as the laser Doppler velocimeter system, phase Doppler anemometry, and particle image velocimetry, it is now possible to separately measure the particle velocity and the fluid velocity in sediment-laden flows (e.g., [5,6,7]). By adopting the laser Doppler velocimeter system, Muste and Patel [5] observed that the stream-wise velocity of suspended sediment 0.23 mm in diameter was less than that of water by as much as 4% in open-channel flows. In the experiment of Muste and Patel [5], the aspect ratios of the employed open-channel flows ranged from 7.05 to 7.11, and the shear velocity estimated from the measured Reynolds shear stress ranged from 3.02 to 3.13 cm/s, respectively. In their experiment, no ripples were formed on the channel bed. Using phase Doppler anemometry, Best et al. [6] adopted phase Doppler anemometry to measure the mean and turbulent characteristics of the water and glass spheres 0.22 mm in mean diameter, and reported that the particle Reynolds number based on the velocity lag ranges from 1 to 5 and the velocity lag increases closer to the channel bed. The experiment by Best et al. [6] was similar to Muste and Patel [5], but the employed particles were glass spheres. Two groups of experiments were performed under the same conditions of shear velocity and water depth, and one group of experiments was conducted under the condition of maximum sediment transport. Similar experimental results regarding velocity lag have been reported in Rashidi et al. [7] in measuring sediment particles in open-channel flows with a water depth of 1.75 cm and Taniere et al. [8] in tracking solid particles in a wind tunnel. Kiger and Pan [9] adopted an image separation technique to measure the particle phase velocity and the fluid phase velocity in pressurized channel flow and showed that the particle lags the mean stream-wise velocity.

In addition to these limited experimental results, there are some theoretical works to analyze the velocity lag in sediment-laden open-channel flows by adopting two-phase flow theory, such as Chauchat and Guillou [10] and Bombardelli and Jha [11]. By analytically solving a two-phase formulation of suspended sediment transport with a hypothesized form of the vertical turbulent intensities and dilute sediment concentrations, Greimann et al. [12] derived velocity lag as a function of the settling velocity and vertical distance above the channel bed. Their formula showed that velocity lag decreases towards the free surface of open-channel flow. A similar two-phase analysis of the velocity lag was carried out by Jiang et al. [13]. Based on the drag force on a sediment particle in the presence of other neighbors, Cheng [1] analytically derived the expression for velocity lag by relating the hindrance coefficient to the shear stress distribution in uniform sediment-laden open-channel flows. Their formula showed that the vertical distribution of velocity lag is related to the shear Reynolds number, particle diameter, and specific gravity of the particle. Furthermore, based on a theoretical analysis of hindered drag force on particle, impact shear stress, viscous shear stress, and turbulent shear stress, Pal et al. [14] formulated a mathematical model for velocity lag as a function of particle diameter, mass density of the particle, friction velocity of the flow, and vertical distance above the channel bottom.

These experimental and theoretical works have provided some valuable physical insights into the physical mechanism of particle-fluid velocity lag of two-phase flow transport in open-channel flows. These works were deterministic. However, in hydraulic engineering studies, there exist always some uncertainties related to some variables and model parameters, such as flow velocity and sediment concentration, due to both the inherent randomness of the turbulent flow and limitations in experiments. Therefore, it is worthy to investigate some hydraulic engineering problems from the viewpoints of the probability method. Currently, few efforts devoted to the study of particle-fluid velocity lag in sediment-laden open-channel flows adopt the probability method based on the entropy theory, except for the work of Kumbhakar et al. [15]. Kumbhakar et al. [15] are the first to adopt the Shannon entropy theory to estimate the velocity lag between a particle and the fluid in sediment-laden open-channel turbulent flows. This motivates us to explore the possibility of another more general entropy function, Tsallis entropy, to predict the stream-wise velocity lag in open-channel flows. As Singh et al. [16] have reviewed, the Tsallis entropy theory together with the principle of maximum entropy have been widely applied to solve certain typical water and environmental engineering problems. For example, the Tsallis entropy theory has been adopted by many researchers to estimate the one-dimensional and two-dimensional velocity distributions of open channels [17,18,19,20], the potential rate of infiltration in unsaturated soils [21,22,23], the vertical distribution of suspended sediment concentration [24,25], the flow–duration curve [26], the sediment concentration distribution in debris flow [27,28], and the thickness of the bed-load layer in an open channel [29]. In these studies, the Tsallis entropy-based model has showed a high prediction accuracy with experimental data, suggesting that the probability method based on the Tsallis entropy theory could be a good addition to some existing deterministic models for approaching certain water and environmental engineering problems [16]. Thus, this study attempts to derive an analytical expression for the stream-wise velocity lag between the particle and the fluid in sediment-laden open-channel flows based on the Tsallis entropy theory together with the principle of maximum entropy. The derived Tsallis entropy-based model is tested against the twenty-two experimental datasets available in the literature, with some deterministic models and the developed Shannon entropy-based model derived by Kumbhakar et al. [15]. This study shows the potential of the Tsallis entropy theory to predict the stream-wise velocity lag between a particle and the surrounding fluid in open channel flows.

## 2. Tsallis Entropy for Velocity Lag

As shown by Figure 1, the objective of this study was to determine the vertical distribution of velocity lag between the sediment particle and the surrounding fluid in sediment-laden flows by adopting the probability method based on the Tsallis entropy theory. Assigning the time-averaged stream-wise velocity lag (or velocity difference) between the fluid and the sediment particles in sediment-laden open-channel flows, $u_{r}\left(y\right)$, at a vertical distance y from the bottom of the open channel, to be a random variable, this study endeavored to formulate the expression for velocity lag between the fluid and the sediment particles in open-channel flows using the Tsallis entropy theory together with the principle of maximum entropy. This included the following six steps: (1) defining the Tsallis entropy function; (2) determining constraint conditions; (3) maximizing the entropy function; (4) determining the Lagrange multipliers; (5) generating a hypothesis for a cumulative distribution function; (6) deriving the velocity lag; and (7) re-parameterizing.

### 2.1. Definition of the Tsallis Entropy

Some experimental studies have shown that velocity lag varies monotonically along the vertical direction in sediment-laden open-channel flows [7,30]. Thus, it can be assumed that velocity lag increases monotonically from zero at the water surface to a maximum value $u_{r\max}$ at a reference position above the channel bottom (at y=a). Therefore, the Tsallis entropy function for the velocity lag in open channel flows, $H(u_{r}\left(y\right))$, can be expressed as [31]

(1) $$H\left(\;u_{r}\left(y\right)\right)=\frac{1}{m-1}\left\{1-\int_{0}^{u_{r\max}}{\left[f\left(u_{r}\right)\right]}^{m}du_{r}\right\}$$

where $f\left(u_{r}\right)$ is the probability density function of velocity lag $u_{r}\left(y\right)$, m is the index for the Tsallis entropy (it is a real number not equal to 1), and $H(u_{r}\left(y\right))$ is the entropy function of $u_{r}\left(y\right)$ or $f\left(u_{r}\right)$. Equation (1) expresses a measure of the uncertainty of $f\left(u_{r}\right)$. Theoretically, the Tsallis entropy function attains a maximum value when $f\left(u_{r}\right)$ is uniform within its limits [16].

### 2.2. Constraint Equations

The probability density function should satisfy the total probability law, that is

(2) $$\int_{0}^{u_{r\max}}f\left(u_{r}\right)du_{r}=1$$

Another constraint equation for $f\left(u_{r}\right)$ is the mean constraint, as follows

(3) $$\int_{0}^{u_{r\max}}u_{r}f\left(u_{r}\right)du_{r}=\bar{u_{r}}$$

where $\bar{u_{r}}$ is the mean (or average) value of $u_{r}\left(y\right)$ along the vertical direction in the sediment-laden open-channel flow [27].

### 2.3. Maximization of Entropy

There are many forms of the probability density function satisfying the constraint equations (Equations (2) and (3)). To choose among all of the probability density functions that satisfy the constraint equations, the maximum entropy principle developed by Jaynes [32,33,34] was used in this study. A method for maximizing the function was to adopt the Euler–Lagrange calculus technique [16]. Therefore, we can construct the Lagrangian function L as follows

(4) $$\begin{array}{l} L=\frac{1}{m-1}\left\{1-\int_{0}^{u_{r\max}}{\left[f\left(u_{r}\right)\right]}^{m}du_{r}\right\}-\lambda_{1}\left[\int_{0}^{u_{r\max}}f\left(u_{r}\right)du_{r}-1\right]\\ -\lambda_{2}\left[\int_{0}^{u_{r\max}}u_{r}f\left(u_{r}\right)du_{r}-\bar{u_{r}}\right] \end{array}$$

where $\lambda_{1}$ and $\lambda_{2}$ are two Lagrange multipliers to be determined from the constraint conditions. Taking $u_{r}$ as an independent variable and $f\left(u_{r}\right)$ as a dependent variable, the Euler–Lagrange equation (Equation (4)) becomes

(5) $$\frac{\partial L}{\partial f}=0\Rightarrow \frac{1}{m-1}\left\{1-m{\left[f\left(u_{r}\right)\right]}^{m-1}\right\}-\lambda_{1}-\lambda_{2}u_{r}=0$$

leading to the expression for $f\left(u_{r}\right)$ as follows

(6) $$f\left(u_{r}\right)={\left[\frac{m-1}{m}\left(\frac{1}{m-1}-\lambda_{1}-\lambda_{2}u_{r}\right)\right]}^{\frac{1}{m-1}}$$

Integrating Equation (6) from 0 to $u_{r}\left(y\right)$ yields the cumulative distribution function for stream-wise velocity lag between the fluid and the sediment particles in sediment-laden open-channel flows, $F\left(u_{r}\right)$, as

(7) $$\begin{array}{l} F\left(u_{r}\right)=\int_{0}^{u_{r}}f\left(u_{r}\right)du_{r}\\ ={\left(\frac{m-1}{m}\right)}^{\frac{m}{m-1}}\frac{1}{\lambda_{2}}\left[{\left(\frac{1}{m-1}-\lambda_{1}\right)}^{\frac{m}{m-1}}-{\left(\frac{1}{m-1}-\lambda_{1}-\lambda_{2}u_{r}\right)}^{\frac{m}{m-1}}\right] \end{array}$$

By substituting Equation (6) into Equation (1), the maximum entropy function $H\left(u_{r}\right)$ becomes

(8) $$H\left(u_{r}\right)=\frac{1}{m-1}\left\{\begin{array}{l} u_{r\max}+{\left(\frac{m-1}{m}\right)}^{\frac{m}{m-1}}\frac{1}{\left(2m-1\right)}\frac{1}{\lambda_{2}}\\ {}*\left[{\left(\frac{1}{m-1}-\lambda_{1}-\lambda_{2}u_{r\max}\right)}^{\frac{2m-1}{m-1}}-{\left(\frac{1}{m-1}-\lambda_{1}\right)}^{\frac{2m-1}{m-1}}\right] \end{array}\right\}$$

### 2.4. Determination of Lagrange Multipliers

Substituting Equation (6) into Equation (2) and integrating lead to

(9) $$\frac{1}{\lambda_{2}}{\left(\frac{m-1}{m}\right)}^{\frac{m}{m-1}}\left[{\left(\frac{1}{m-1}-\lambda_{1}\right)}^{\frac{m}{m-1}}-{\left(\frac{1}{m-1}-\lambda_{1}-\lambda_{2}u_{r\max}\right)}^{\frac{m}{m-1}}\right]=1$$

Substituting Equation (6) into Equation (3) and integrating yield

(10) $$\begin{array}{l} u_{r\max}{\left(\frac{1}{m-1}-\lambda_{1}-\lambda_{2}u_{r\max}\right)}^{\frac{m}{m-1}}+\frac{m-1}{2m-1}\frac{1}{\lambda_{2}}\\ {}*\left[{\left(\frac{1}{m-1}-\lambda_{1}-\lambda_{2}u_{r\max}\right)}^{\frac{2m-1}{m-1}}-{\left(\frac{1}{m-1}-\lambda_{1}\right)}^{\frac{2m-1}{m-1}}\right]+\lambda_{2}\bar{u_{r}}{\left(\frac{m}{m-1}\right)}^{\frac{m}{m-1}}=0 \end{array}$$

Equations (9) and (10) constitute a non-linear equation system for two Lagrange multipliers, $\lambda_{1}$ and $\lambda_{2}$.

### 2.5. Hypothesis for the Cumulative Distribution Function of Velocity Lag

To formulate the expression for velocity lag in the real (space) domain, a hypothesis regarding the cumulative distribution function of velocity lag that connects the probability domain to the space domain needs to be made [16,35]. The hypothesized cumulative distribution function of velocity lag should satisfy the following characteristics: (a) it is a continuous and differentiable function; (b) it varies from 0 to 1; (c) it takes the maximum value 1 at the reference position, and the zero value at the water surface of open-channel flow. Meanwhile, it should be able to reflect some characteristics of the vertical distributions of the flow and sediment particle concentrations in sediment-laden open-channel flows. Kumbhakar et al. [15] proposed the following power-type hypothesis regarding the cumulative distribution function $F(u_{r}\left(y\right))$ to be a good choice

(11) $$F(u_{r}\left(y\right))=1-{\left(\frac{y-a}{H-a}\right)}^{\eta }$$

that showed a good agreement with some experimental results. In this equation, H is the flow depth and η is a fitting parameter. Kumbhakar et al. [15] showed that the hypothesis for the cumulative distribution function, Equation (11), is based on two significant assumptions: (a) all values of y between 0 and H are equally likely, and (b) the velocity lag increases monotonically from the water surface of the open-channel flow to the reference position.

### 2.6. Derivation of Velocity Lag

Equating Equations (7) and (11), we can obtain the stream-wise velocity lag between the fluid and a sediment particle in sediment-laden open-channel flows $u_{r}\left(y\right)$, as

(12) $$\begin{array}{l} u_{r}\left(y\right)=-\frac{1}{\lambda_{2}}{\left\{{\left(\frac{1}{m-1}-\lambda_{1}\right)}^{\frac{m}{m-1}}-\lambda_{2}{\left(\frac{m}{m-1}\right)}^{\frac{m}{m-1}}\left[1-{\left(\frac{y-a}{H-a}\right)}^{\eta }\right]\right\}}^{\frac{m-1}{m}}\\ -\frac{\lambda_{1}}{\lambda_{2}}+\frac{1}{\lambda_{2}\left(m-1\right)} \end{array}$$

### 2.7. Reparameterization

Letting $\lambda_{*}=\frac{1}{m-1}-\lambda_{1}$, Equation (12) becomes

(13) $$\begin{array}{l} u_{r}=\frac{\lambda_{*}}{\lambda_{2}}-\frac{1}{\lambda_{2}}\frac{m}{\left(m-1\right)}{\left\{{\left(\frac{m-1}{m}\lambda_{*}\right)}^{\frac{m}{m-1}}-\lambda_{2}\left[1-{\left(\frac{y-a}{H-a}\right)}^{\eta }\right]\right\}}^{\frac{m-1}{m}} \end{array}$$

Similar to Singh and Cui [27], we defined non-dimensional entropy parameter G as follows, $G=\frac{\lambda_{2}u_{r\max}}{\lambda_{2}u_{r\max}-\lambda_{*}}$, in this study. Dividing Equation (13) by $u_{r\max}$, we can obtain the velocity lag in terms of the non-dimensional entropy parameter as follows

(14) $$\frac{u_{r}}{u_{r\max}}=1-\frac{1}{G}-{\left\{{\left(1-\frac{1}{G}\right)}^{\frac{m}{m-1}}-{\left(\frac{m}{m-1}\right)}^{\frac{1}{m-1}}\lambda_{1}^{\frac{1}{1-m}}u_{r\max}^{\frac{m}{1-m}}\left[1-{\left(\frac{y-a}{H-a}\right)}^{\eta }\right]\right\}}^{\frac{m-1}{m}}$$

At the reference position (i.e., y=a), we have $F(u_{r}\left(a\right))=1$, and $u_{r}\left(y\right)$ takes the maximum value $u_{r\max}$. Thus, we can obtain from Equation (14)

(15) $$\lambda_{1}^{\frac{1}{1-m}}u_{r\max}^{\frac{m}{1-m}}=\frac{{\left(1-\frac{1}{G}\right)}^{\frac{m}{m-1}}-{\left(-\frac{1}{G}\right)}^{\frac{m}{m-1}}}{{\left(\frac{m}{m-1}\right)}^{\frac{1}{m-1}}}$$

By substituting Equation (15) into Equation (14), the velocity lag can be written in terms of the non-dimensional entropy parameter as

(16) $$\frac{u_{r}}{u_{r\max}}=1-\frac{1}{G}+\frac{1}{G}{\left\{{\left(1-G\right)}^{\frac{m}{m-1}}+\left[1-{\left(1-G\right)}^{\frac{m}{m-1}}\right]*\left[1-{\left(\frac{y-a}{H-a}\right)}^{\eta }\right]\right\}}^{\frac{m-1}{m}}$$

When normalized by the shear velocity, $u_{*}$, the non-dimensional velocity lag in this equation can be expressed as

(17) $$\frac{u_{r}}{u_{*}}=\left(1-\frac{1}{G}\right)\frac{u_{r\max}}{u_{*}}+\frac{1}{G}\frac{u_{r\max}}{u_{*}}{\left\{{\left(1-G\right)}^{\frac{m}{m-1}}+\left[1-{\left(1-G\right)}^{\frac{m}{m-1}}\right]*\left[1-{\left(\frac{y-a}{H-a}\right)}^{\eta }\right]\right\}}^{\frac{m-1}{m}}$$

**Figure 2:**
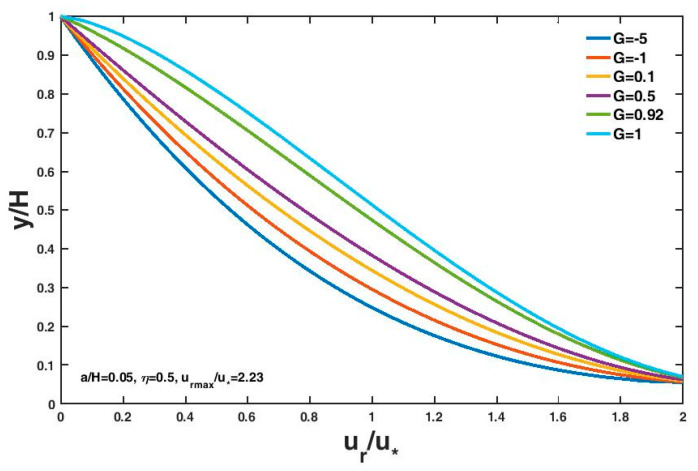
Variation of the non-dimensional velocity lag with vertical distance from the channel bottom with different G values.

Equation (17) denotes the Tsallis entropy-based expression for the non-dimensional stream-wise velocity lag between the fluid and a sediment particle in sediment-laden open-channel flows. The vertical distribution of the non-dimensional velocity lag with different G values is shown in Figure 2. The parameter values were taken from the experimental results of Rashida et al. [7]: a=0.05H, η=0.5 and $u_{r\max}=2.23u_{*}$. Different G values lead to different velocity lag distributions, and as G tends to 0.92, $\frac{u_{r}}{u_{*}}$ approximately decreases linearly with $\frac{y}{h}$. The non-dimensional entropy parameter G can be regarded as a measure of the uniformity of the velocity lag distribution. At a fixed vertical position from the channel bottom, the velocity lag between the fluid and the sediment particle in open-channel flows increases with increasing G value.

## 3. Comparison with Experimental Data and Other Models and Discussion

### 3.1. Selected Experimental Data

There are few experimental results regarding stream-wise velocity lag between the fluid and a particle in open-channel flows in the literature. Six datasets of experimental results available from the literature collected by Pal et al. [14] and Kumbhakar et al. [15] were adopted in this study to test the validity of the Tsallis entropy-based expression of velocity lag (Equation (17)). These datasets included Rashidi et al. [7], Kaftori et al. [36], Best et al. [6], Muste and Patel [5], Righetti and Romano [30], and Muste et al. [37]. Table 1 lists the hydraulic conditions of these twenty-two collected datasets. The third column shows the particle material adopted in the experiments. The particle diameter d, the particle specific gravity s (= $\frac{\rho_{s}}{\rho_{f}}$, where $\rho_{s}$ and $\rho_{f}$ are densities of the particle and the fluid, respectively), the shear velocity $u_{*}$ of the flow, the flow depth H, and the kinematic viscosity of the fluid $\nu_{f}$ are presented in the fourth, fifth, sixth, seventh, and eighth columns, respectively. Using the shear velocity, the particle diameter and the kinematic viscosity of the fluid, we can estimate the Reynolds number Re∗ (= $\frac{u_{*}d}{\nu_{f}}$) for each case as listed in the last column. This table shows that the collected datasets contain different particle materials: polystyrene, glass or natural sand, and cover a wide range of flow condition Re∗, from low flow conditions (Re∗ = 1.29, 1.6, 1.98) to high flow conditions (Re∗ = 11.79, 14.89, 17.89).

To evaluate the accuracy of the proposed model and other models with the collected experimental datasets, an error analysis was adopted in this study by calculating the average value of the relative error R in percent, as follows

(18) $$R\;=\;\frac{100}{N}\left[\sum_{i=1}^{N}\left|\frac{{\left(\frac{u_{r}}{u_{*}}\right)}_{i}^{m}-{\left(\frac{u_{r}}{u_{*}}\right)}_{i}^{o}}{{\left(\frac{u_{r}}{u_{*}}\right)}_{i}^{o}}\right|\right]$$

where ${\left(\frac{u_{r}}{u_{*}}\right)}_{i}^{m}$ and ${\left(\frac{u_{r}}{u_{*}}\right)}_{i}^{o}$ are the modelled and observed non-dimensional velocity lag values, respectively, and N is the total number of points in the dataset. The fitting accuracy improves as R decreases. Relative error analysis has been frequently adopted and confirmed to be a good statistical method to compare the prediction accuracy of the developed models by some researchers [15,17,18,19,20,24,25,27,28,29].

### 3.2. Some Deterministic Models

Three kinds of deterministic models for the stream-wise velocity lag in open-channel flows were collected in this study and presented as follows. By analytically solving the two-phase formulation of suspended sediment transport with a hypothesized form of the vertical turbulent intensities and dilute sediment concentrations, Greimann et al. [12] derived the velocity lag expression as

(19) $$\frac{u_{r}}{u_{*}}=0.66\frac{\omega }{u_{*}}\left(1-\frac{y}{H}\right)\exp\left(1.34\frac{y}{H}\right)$$

where ω is the settling velocity of a sediment particle. In this study, the following widely cited formula by Cheng [38], as adopted in Pal et al. [14], $\omega =\frac{\nu_{f}}{d}{\left(\sqrt{25+1.2*{\left[\frac{\left(s-1\right)g}{\nu_{f}^{2}}\right]}^{\frac{2}{3}}d^{2}}-5\right)}^{1.5}$, where g is the gravitational acceleration, was adopted to estimate the particle’s settling velocity.

Based on the drag force on a sediment particle in the presence of other neighbors, Cheng [1] analytically derived the expression for velocity lag by relating the hindrance coefficient to the shear stress distribution in uniform sediment-laden open-channel flows as follows

(20) $$\frac{u_{r}}{u_{*}}={\left[\sqrt{{\left(2-2\frac{y}{H}\right)}^{\frac{1}{1.5}}+\frac{1}{4}{\left(32\frac{\nu_{f}}{u_{*}d}\right)}^{\frac{2}{1.5}}}-\frac{1}{2}{\left(32\frac{\nu_{f}}{u_{*}d}\right)}^{\frac{1}{1.5}}\right]}^{1.5}$$

Furthermore, based on a theoretical analysis of hindered drag force on a particle, impact shear stress, viscous shear stress, and turbulent shear stress, Pal et al. [14] formulated a mathematical model for velocity lag as

(21) $$\frac{u_{r}}{u_{*}}={\left[\sqrt{{\left(\frac{M}{1.17}\right)}^{\frac{1}{1.75}}+\frac{1}{4}{\left(\frac{N}{1.17}\right)}^{\frac{2}{1.75}}}-\frac{1}{2}{\left(\frac{N}{1.17}\right)}^{\frac{1}{1.75}}\right]}^{1.75}$$

where M and N are given by

(22) $$M=\frac{2\tau_{a}}{\rho_{m}u_{*}^{2}}$$

(23) $$N=32.2\mu_{r}{\left(1-c\right)}^{s-1}\frac{\nu_{f}}{u_{*}d}$$

here

(24) $$\tau_{a}=\frac{\alpha_{\tau }\rho_{f}u_{*}^{2}}{d}\int_{y}^{1}\left[B_{1}+B_{2}+\left(1-\frac{y}{H}\right)\left(1-c^{\frac{1}{3}}\right)\right]dy$$

(25) $$B_{1}=\frac{s*c^{\frac{2}{3}}}{(1-c^{\frac{1}{3}}{)}^{2}}*\frac{d^{2}}{k_{m}^{2}}*[y+\prod \pi H\sin\left(\pi \frac{y}{H}\right){]}^{2}$$

(26) $$B_{2}=\frac{\mu_{r}*c^{\frac{2}{3}}}{\left(1-c^{\frac{1}{3}}\right)}*\frac{\nu_{f}}{u_{*}k_{m}}*\left[y+\prod \pi H\sin\left(\pi \frac{y}{H}\right)\right]$$

In Equations (21)–(26), $\rho_{m}$ is the mass density of the sediment-fluid mixture, $\mu_{r}$ is the ratio of the dynamic viscosities of the sediment-laden mixture and the sediment-free fluid, c is the volumetric concentration of sediment particles, $\alpha_{\tau }$ is the proportionality parameter introduced as the velocity lag coefficient, $k_{m}$ is the von Karman constant of the sediment-fluid mixture, and ∏ is the wake parameter varying with c, respectively.

Regarding $\alpha_{\tau }$, Pal et al. [14] obtained the following formula by a regression analysis,

(27) $$\alpha_{\tau }=618.741s^{2.45}{\left(\frac{u_{*}}{\omega }\right)}^{0.812}{\left(\frac{d}{H}\right)}^{1.896}$$

whereas regarding ∏, they derived the following regression equation for a wide range of particle concentrations: $\prod =1.13\bar{c}+0.34$, where $\bar{c}$ is the average particle concentration.

For these deterministic velocity-lag models (Equations (19)–(21)), more detailed introductions can be found in the studies of Greimann et al. [12], Cheng [1], and Pal et al. [14], respectively.

### 3.3. Comparison with Experimental Data and Other Models

**Figure 3:**
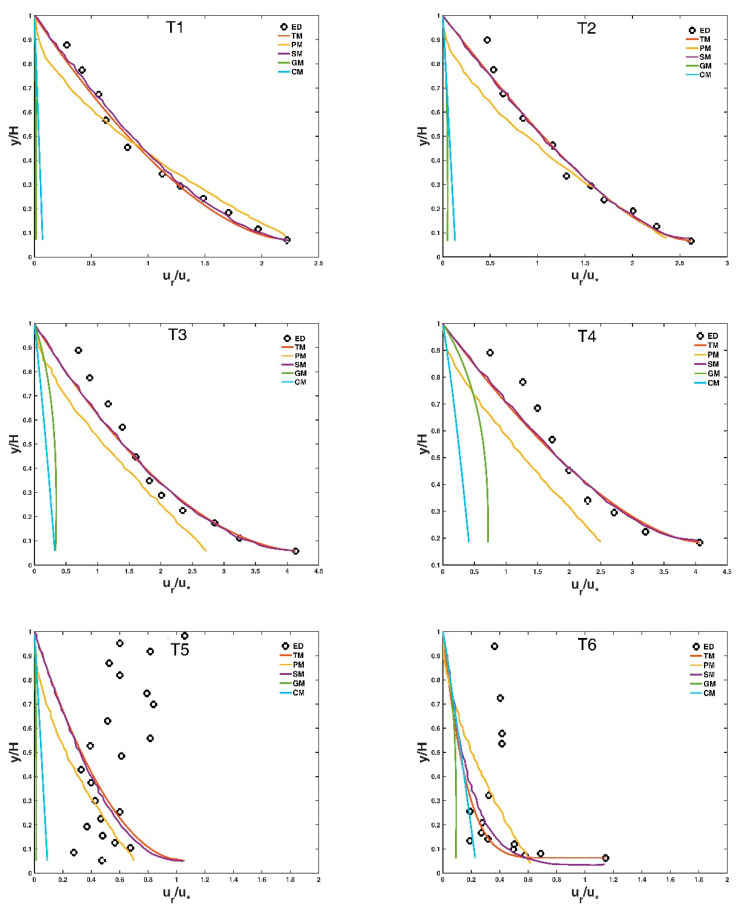

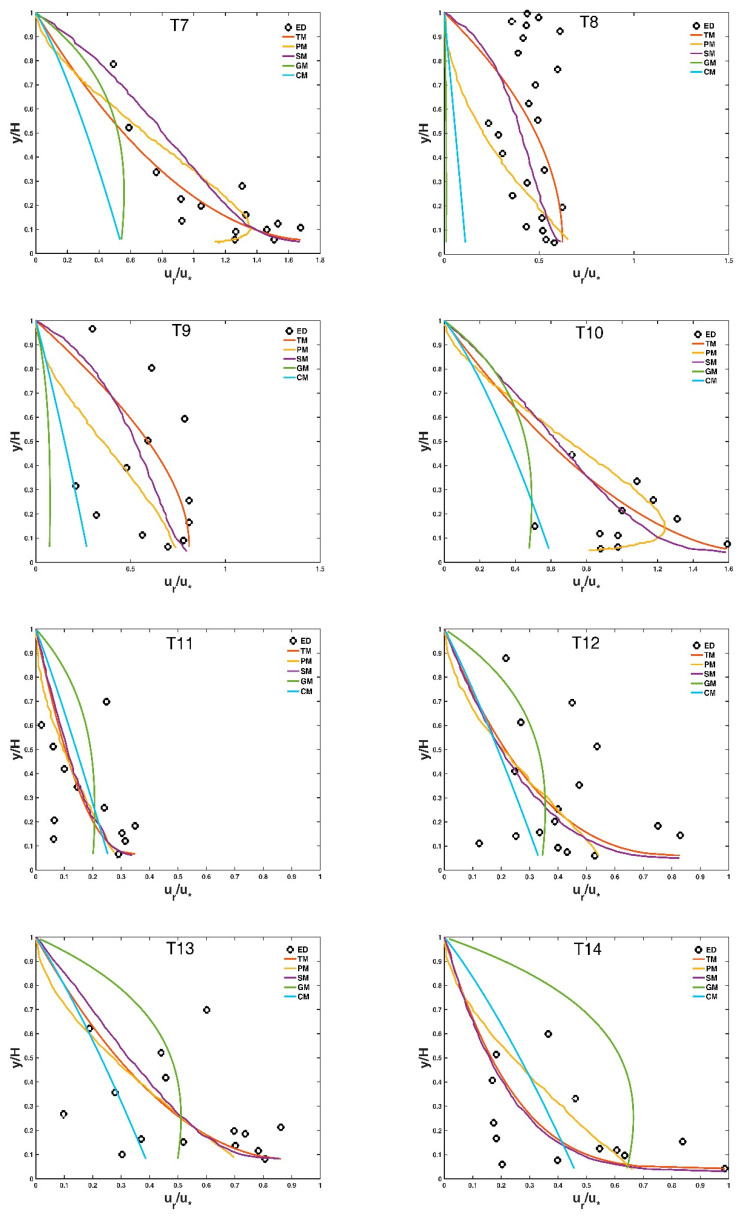

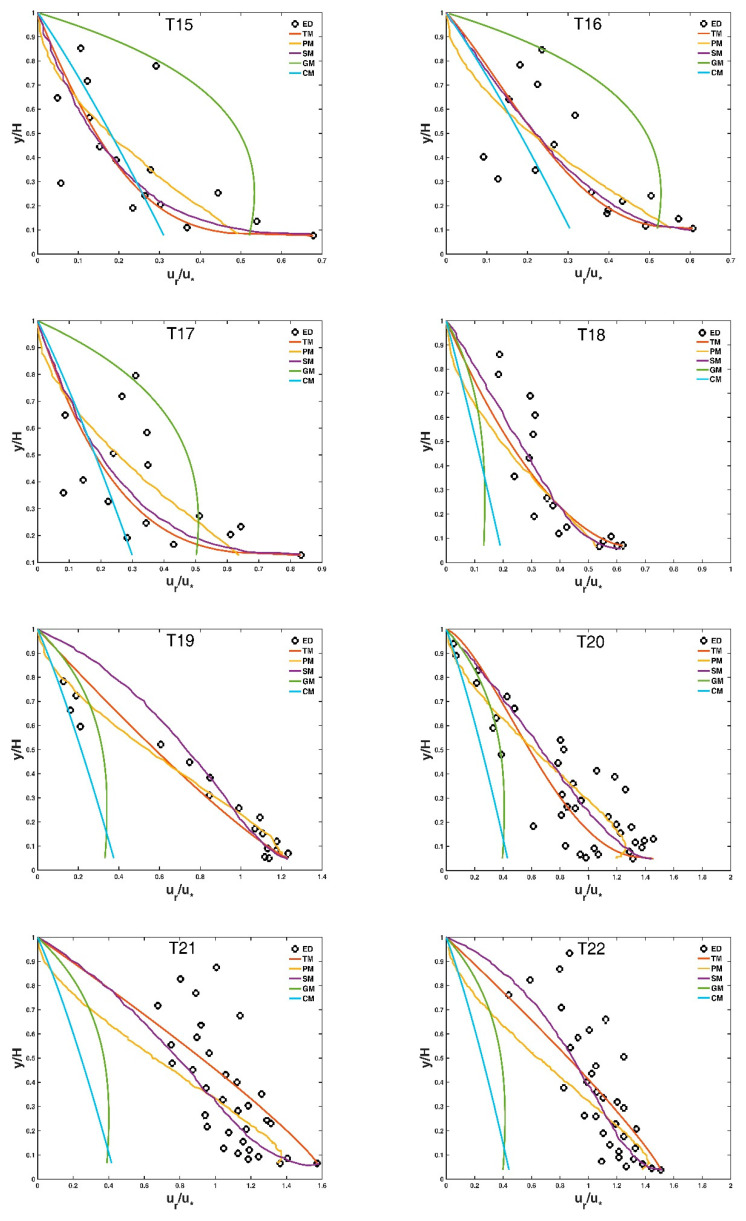
Comparison of the derived expression for the Tsallis entropy (Equation (17)), the deterministic model of Greimann et al. [12] (Equation (19)), the deterministic model of Cheng [1] (Equation (20)), the deterministic model of Pal et al. [14] (Equation (21)), as well as the Shannon entropy-based model derived by Kumbhakar et al. [15], with twenty-two collected experimental datasets (T1–T22). In each figure, ED: experimental data; TM: the Tsallis entropy-based model (Equation (17)); PM: the deterministic model of Pal et al. [14] (Equation (21)); SM: the Shannon entropy-based model derived by Kumbhakar et al. [15]; GM: the deterministic model of Greimann et al. [12] (Equation (19)); CM: the deterministic model of Cheng [1] (Equation (20)).

For each experimental dataset, the two Lagrange multipliers in Equation (17), $\lambda_{1}$ and $\lambda_{2}$, can be computed by solving the non-linear equation system (i.e., Equations (9) and (10)), provided the values of $\bar{u_{r}}$ and $u_{r\max}$ are given from the experimental dataset. The value for the parameter η can be estimated by fitting Equation (11) to the experimental dataset. The entropy index m = 3, as adopted in Singh and Cui [27], was used in this study. Figure 3 presents the comparison of the proposed Tsallis entropy-based model (Equation (17)), the deterministic model of Greimann et al. [12], (Equation (19)), the deterministic model of Cheng [1] (Equation (20)), the deterministic model of Pal et al. [14] (Equation (21)), as well as the Shannon entropy-based model derived by Kumbhakar et al. [15], with twenty-two collected experimental datasets. Table 2 presents the calculated R values for each case by five different models. In this table, the symbol *** in each row denotes the minimum error for each case. For eighteen out of the twenty-two total test cases, the entropy-based models (Equation (17) or the Shannon entropy-based model derived by Kumbhakar et al. [15]) have obviously better prediction accuracies than the other three deterministic models, except for T5, T12, T19 and T20. In twelve out of eighteen test cases (T1, T2, T3, T4, T6, T7, T11, T13, T14, T15, T16, T17), the Tsallis entropy-based model (Equation (17)) agrees slightly better with the experimental data points than the Shannon entropy-based model, whereas for the other six test cases (T8, T9, T10, T18, T21, T22), the Shannon entropy-based model has a slightly higher prediction accuracy. Considering that the calculated R values for the Tsallis entropy-based model are close to those of the Shannon entropy-based model, and that there is data scattering (as shown in Figure 3), as well as some experimental uncertainties, it can be concluded that this study shows the potential of the Tsallis entropy theory together with the principle of maximum entropy as well as the developed Shannon entropy-based model to predict the stream-wise velocity lag between a particle and the surrounding fluid in open-channel sediment-laden flows. The Tsallis entropy-based model, as well as the Shannon entropy-based model, are good additions to some existing deterministic models for the prediction of the fluid-particle velocity lag along the stream-wise direction in uniform sediment-laden open channel flows. It should be also noted that for some experimental data sets (e.g., T5, T8, T12, T13), there are serious data scattering, and these data points did not follow any clear trend may be due to the uncertainty of experiment measurements in tracking the velocities of the particle and the fluid and the inherent randomness of the turbulent flow. For those real cases, neither the proposed entropy-based models nor the existing deterministic models can be applicable, and theses indicate the limitations of the proposed models and the existing deterministic models for predicting the velocity lag between the sediment particle and the fluid in sediment-laden open-channel flows.

### 3.4. Physical Explanation

By connecting the fitting parameter η to some known quantities of the sediment-laden flow by virtue of a regression analysis, Kumbhakar et al. [15] showed that the fitting parameter η is proportional to ${\left(s\right)}^{-0.602}{\left(\frac{u_{*}}{\omega }\right)}^{-0.156}{\left(\frac{d}{H}\right)}^{-0.194}$. Based on this mathematical relationship, we attempted to discuss the impacts of some parameters on the derived Tsallis entropy-based model (Equation (16)) by simply choosing the test case T1 as a typical example and varying the mentioned parameter with the other parameter values fixed.

**Figure 4:**
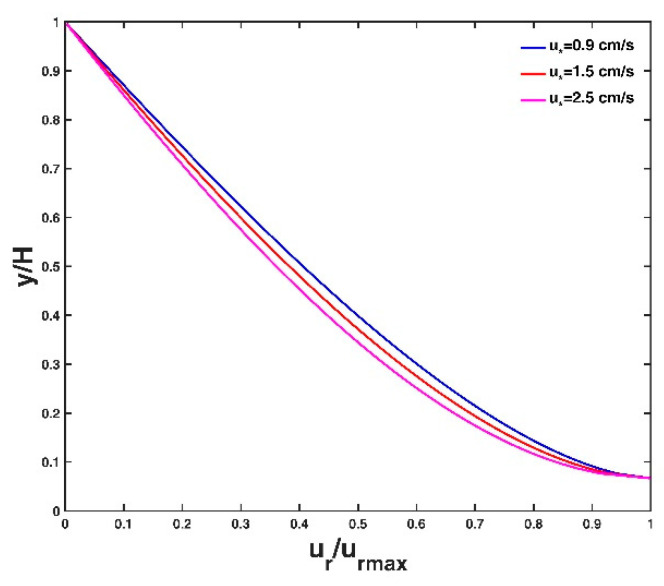
Vertical velocity lag distribution for three different values of friction velocity of the flow. Other parameter values are presented in the test case T1 of Table 1.

**Figure 5:**
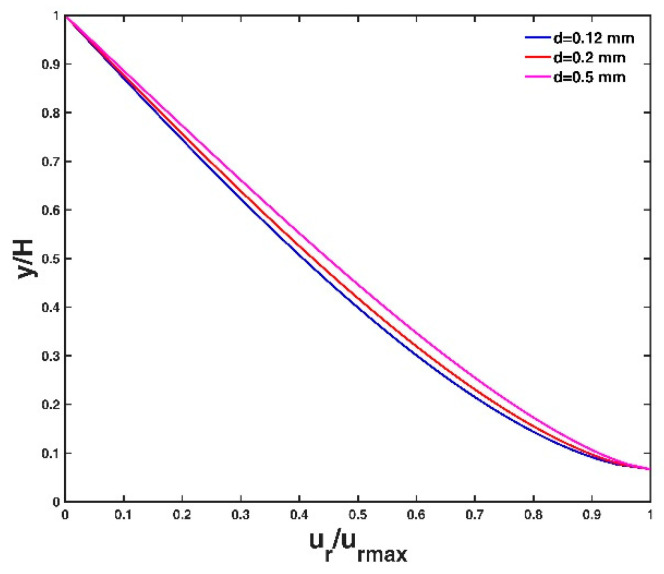
Vertical velocity lag distribution for three different values of particle diameter. Other parameter values are presented in the test case T1 of Table 1.

**Figure 6:**
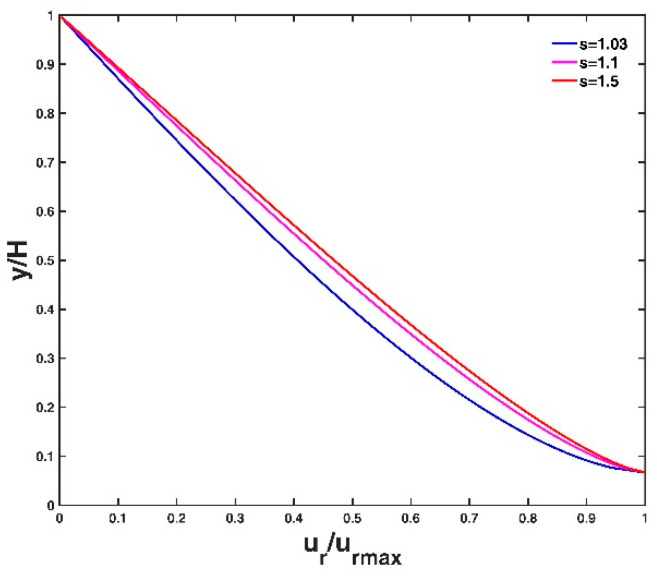
Vertical velocity lag distribution for three different values of particle specific gravity. Other parameter values are presented in the test case T1 of Table 1.

Figure 4 shows the vertical distribution of velocity lag at three different conditions of the friction velocity, $u_{*}$, of the flow. A large flow friction velocity leads to a small velocity lag between the particle and the fluid. This is because a large friction velocity value corresponds to a strong flow shear stress, which can carry the particle well to follow the fluid velocity, and as a result, the velocity lag decreases. This agrees with the analysis results of Pal et al. [14] and Kumbhakar et al. [15]. Figure 5 shows the vertical distribution of velocity lag at three different conditions of particle diameter d. The velocity lag increases with increasing particle diameter. A particle with a large diameter has a large surface area and a larger gravitational force for the same specific gravity, leading to a slower acceleration with the flow velocity, and consequently, the velocity lag increases. The vertical distribution of velocity lag at three different conditions of particle specific gravity s are presented in Figure 6. The velocity lag increases with increasing specific gravity of the particle, which is inconsistent with Kumbhakar et al. [15] but consistent with the analysis result of Pal et al. [14]. A reasonable explanation is similar to the case of varying particle diameter as mentioned above: a particle with a large specific gravity value has a large gravitational force. A heavy particle is difficult for the flow to accelerate in the stream-direction compared to a light particle, thus the velocity lag increases.

The Tsallis entropy-based model (Equation (17)) can estimate the velocity lag between a fluid and a particle along the stream-wise direction in sediment-laden open-channel flows with a high prediction accuracy, as presented in Figure 3, as long as the non-dimensional entropy parameter G depending on the average and maximum values of the measured velocity lag is provided from experimental cases. The Tsallis entropy-based model has a simpler mathematical form in comparison to the deterministic model of Pal et al. [14] (Equation (21)) and it has fewer input parameters compared with the existing deterministic models. The Tsallis entropy-based model, together with the developed Shannon entropy-based model, are good additions to the existing deterministic models to predict the particle-fluid velocity difference along the stream-wise direction in sediment-laden open channel flows. However, some physical properties cannot be directly incorporated into the entropy-based model. For example, the kinematic viscosity of the sediment-laden flow and the particle diameter are two parameters that play important roles in accelerating the particle to follow the fluid velocity in open-channel flows [1,14]. However, the Tsallis entropy-based model does not contain these parameters.

## 4. Concluding Remarks

In this study, a model for estimating velocity lag between a particle and the surrounding fluid along the stream-wise direction in sediment-laden open-channel flows was derived based on the Tsallis entropy theory, together with the principle of maximum entropy. The derived model was expressed in terms of a non-dimensional entropy parameter depending on the average and maximum values of measured velocity lag from the experiment.

The derived Tsallis entropy-based model was tested against twenty-two collected experimental cases from the literature, with three deterministic models and the developed Shannon entropy-based model. The Tsallis entropy-based model agreed better with the experimental datasets than the deterministic models for eighteen out of the twenty-two total real cases, and the prediction accuracy for the eighteen experimental datasets was comparable to that of the developed Shannon entropy-based model (the Tsallis entropy-based expression agreed slightly better than the Shannon entropy-based model for twelve out of eighteen test cases, whereas for the other six test cases, the Shannon entropy-based model had a slightly higher prediction accuracy).

Based on the Tsallis entropy-based model, decreasing the friction velocity of the flow or increasing either the particle diameter or specific gravity leads to an increased velocity lag in open-channel flows. These results are consistent with our physical understanding of two-phase flow in sediment-laden open-channel flows.

This study shows the potential of the Tsallis entropy theory together with the principle of maximum entropy, as well as the developed Shannon entropy-based model, to predict the stream-wise velocity lag between a particle and the surrounding fluid in sediment-laden open-channel flows. The Tsallis entropy-based model, as well as the Shannon entropy-based model, can be good additions to some existing deterministic models for the prediction of the fluid-particle velocity lag in the stream-wise direction in uniform sediment-laden open-channel flows.

## Figures and Tables

**Table 1:** Hydraulic conditions of six experimental datasets collected from the literature.

Test Number	Reference	Particle Material	Particle Diameter d (mm)	Particle Specific Gravity s	$\mathrm{Shear}\;\mathrm{Velocity}\;u_{*}\;(\mathrm{cm}/s)$	Flow Depth H (cm)	$\mathrm{Kinematic}\;\mathrm{Viscosity}\;\mathrm{of}\;\mathrm{the}\;\mathrm{Fluid}\;\nu_{f}\;({\mathrm{cm}}^{2}/s)$	Reynolds NumberRe∗
T1	Rashidi et al. [7]	Polystyrene	0.12	1.03	0.9	2.75	0.0084	1.286
T2			0.22	1.03	0.9	2.75	0.0084	2.357
T3			0.65	1.03	0.9	2.75	0.0084	6.964
T4			1.10	1.03	0.9	2.75	0.0084	11.786
T5	Kaftori et al. [36]	Polystyrene	0.1	1.05	1.28	3.25	0.008	1.600
T6			0.275	1.05	1.29	3.27	0.0079	4.491
T7			0.9	1.05	1.34	3.27	0.0081	14.889
T8			0.1	1.05	1.6	3.52	0.0081	1.975
T9			0.275	1.05	1.6	3.51	0.008	5.500
T10			0.9	1.05	1.55	3.77	0.0078	17.885
T11	Best et al. [6]	Glass	0.125	2.6	3.4	5.75	0.0083	5.120
T12			0.175	2.6	3.4	5.75	0.0083	7.169
T13			0.225	2.6	3.4	5.75	0.0083	9.217
T14			0.275	2.6	3.4	5.75	0.0083	11.265
T15	Muste and Patel [5]	Natural sand	0.23	2.65	3.02	12.9	0.0103	6.744
T16			0.23	2.65	3.05	12.9	0.0103	6.811
T17			0.23	2.65	3.13	12.9	0.0105	6.856
T18	Righetti and Romano [30]	Glass	0.1	2.6	3.29	2.3	0.009	3.656
T19			0.2	2.6	3.97	2	0.0094	8.447
T20	Muste et al. [37]	Natural sand	0.23	2.65	4.2	2.1	0.0093	10.387
T21			0.23	2.65	4.2	2.1	0.0096	10.063
T22			0.23	2.65	4.2	2.1	0.0091	10.615

**Table 2:** Comparison result of the derived expression for the Tsallis entropy (Equation (17)), the Greimann et al. [12] model (Equation (19)), the Cheng [1] model (Equation (20)), the Pal et al. [14] model (Equation (21)), and the Shannon entropy-based model proposed by Kumbhakar et al. [15] with twenty-two collected laboratory datasets. In each row, the symbol *** corresponds to the minimum error for each case.

Test Number	Fitting Result: R Value				
	Tsallis Entropy-Based Model	Greimann et al. [12] Model	Cheng [1] Model	Pal et al. [14] Model	Shannon Entropy-Based Model [15]
T1	8.38 ***	98.48	95.79	21.65	8.69
T2	9.85 ***	96.16	94.04	22.88	9.98
T3	15.03 ***	82.86	89.46	32.68	15.51
T4	17.60 ***	69.98	87.53	42.75	17.96
T5	63.44	97.81	89.16	57.24 ***	59.55
T6	41.52 ***	75.94	53.04	59.21	44.08
T7	18.28 ***	49.08	59.15	18.43	18.52
T8	47.92	98.23	87.37	54.29	42.39 ***
T9	56.02	87.02	63.59	52.88	50.36 ***
T10	36.91	47.88	44.83	44.42	33.70 ***
T11	90.82 ***	147.92	115.36	94.38	97.19
T12	68.09	38.92 ***	48.46	58.20	60.81
T13	61.13 ***	65.69	61.79	61.96	62.07
T14	54.16 ***	117.68	57.86	69.26	55.78
T15	46.69 ***	193.70	56.01	67.96	50.00
T16	38.45 ***	90.44	47.19	50.31	40.94
T17	50.96 ***	104.97	51.73	71.01	55.60
T18	25.17	65.54	64.08	30.31	21.68 ***
T19	28.52	61.72	57.65	15.71 ***	49.53
T20	30.85	52.87	59.14	26.30 ***	26.64
T21	22.45	65.27	72.40	26.31	18.60 ***
T22	19.80	65.23	72.08	27.08	12.23 ***
